# Supplementary material for: Splice-Junction-Based Mapping of Alternative Isoforms in the Human Proteome
Source: Cell Rep. Author manuscript; Available in PMC 2020 Jan 15. (PMC6961840; doi:10.1016/j.celrep.2019.11.026)
Supplement: 2 [file NIHMS1546469-supplement-2.zip › DF1/PXD010154/Testis/Testis_15_STK39_EENPEGPPNANEDYR.pdf]

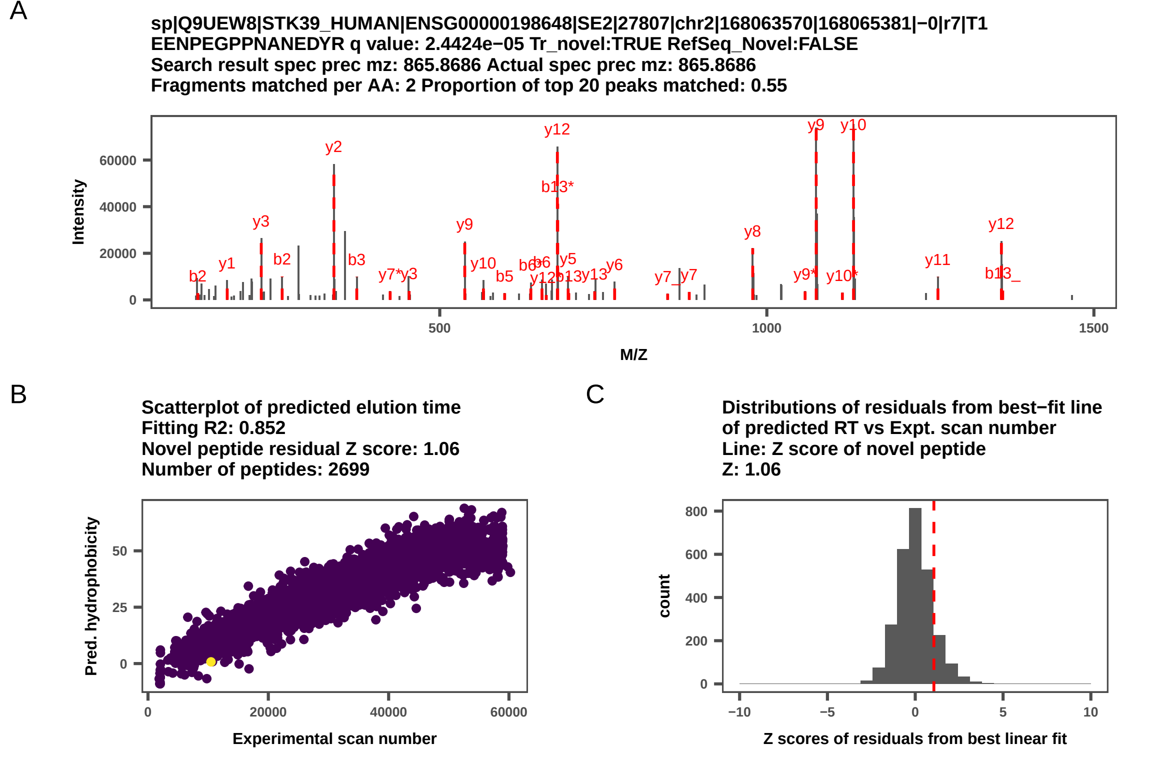
A
sp|Q9UEW8|STK39_HUMAN|ENSG00000198648|SE2|27807|chr2|168063570|168065381|−0|r7|T1
EENPEGPPNANEDYR q value: 2.4424e−05 Tr_novel:TRUE RefSeq_Novel:FALSE
Search result spec prec mz: 865.8686 Actual spec prec mz: 865.8686
Fragments matched per AA: 2 Proportion of top 20 peaks matched: 0.55
Intensity
0
20000
40000
60000
M/Z
500
1000
1500
y12
y2
b13*
y9
y10
y3
y9
y8
y12
y1
b2
b2
b3
y7*
y3
y10
b5
b6*
b6
y5
y12
b13
y13
y6
y7_
y7
y9*
y10*
y11
b13_
B
Scatterplot of predicted elution time
Fitting R2: 0.852
Novel peptide residual Z score: 1.06
Number of peptides: 2699
Pred. hydrophobicity
0
25
50
Experimental scan number
0
20000
40000
60000
C
Distributions of residuals from best−fit line
of predicted RT vs Expt. scan number
Line: Z score of novel peptide
Z: 1.06
count
0
200
400
600
800
Z scores of residuals from best linear fit
−10
−5
0
5
10
